# Gastroprotective effect of the traditional herbal medicine, Sipjeondaebo-tang water extract, against ethanol-induced gastric mucosal injury

**DOI:** 10.1186/1472-6882-14-373

**Published:** 2014-10-04

**Authors:** Woo-Young Jeon, In-Sik Shin, Hyeun-Kyoo Shin, Mee-Young Lee

**Affiliations:** Herbal Medicine Formulation Research Group, Korea Institute of Oriental Medicine, 483 Expo-ro, Yusung-gu, Daejeon, 305-811 South Korea; Natural Medicine Research Center, Korea Research Institute of Bioscience and Biotechnology, 30 Yeongudanji-ro, O-chang uep, Cheongwon-gun, Chungbuk, 363-883 South Korea

**Keywords:** Sipjeondaebo-tang, Gastric mucosal injury, Malondialdehyde, Glutathione, Antioxidant enzymes

## Abstract

**Background:**

Sipjeondaebo-tang, a traditional herbal medicine, has been reported to activate the immune response. Although, most research has focused on its anticancer activity. The purpose of this study was to determine whether Sipjeondaebo-tang exerts antioxidant activity against ethanol-induced gastric injury.

**Methods:**

Gastric mucosal injury was induced by the oral administration of absolute ethanol at 5 mL/kg to rats after 18 h fast. Sipjeondaebo-tang water extract (SDTW; 200 mg/kg of body weight) was administered to rats 2 h before the oral administration of absolute ethanol. Gastric mucosal injury was evaluated by measuring the gastric injury, such as extent of lesions, malondialdehyde (MDA) concentration, glutathione (GSH) content and activities of antioxidant enzymes including catalase, glutathione peroxidase, glutathione S-transferase, glutathione reductase, and superoxide dismutase in stomach tissue.

**Results:**

Oral administration of SDTW markedly decreased the damage by conditioning the gastric mucosa such as hemorrhage, hyperemia. Pretreatment with SDTW significantly reduced MDA concentration and significantly increased GSH content and the activities of antioxidant enzymes. In an acute toxicity study, no adverse effects of SDTW were observed at doses up to 5000 mg/kg/day.

**Conclusions:**

SDTW may protect the gastric mucosa against ethanol-induced gastric mucosa injury. These results suggested that SDTW might also play an important role in the gastroprotection based on their antioxidant effect.

## Background

Ethanol has been shown to cause damage to the gastric mucosa in both animal and clinical studies, mainly by inducing severe gastric hemorrhagic lesions. Thus, an experimental model of ethanol-induced gastric mucosal injury in rats is often used for screening agents with potential gastroprotective effects [1].

Ethanol intake is associated with marked oxidative damage to the gastric mucosa and induces the overproduction of reactive oxygen species (ROS), the main mediators of oxidative stress, and decreases the activities of antioxidant enzymes [2]. Previous study has demonstrated that ethanol-induced gastric lesions are closely related to increased ROS levels, which lead to lipid peroxidation in the membranes through the oxidation of unsaturated fatty acids [3]. A number of studies have investigated the gastroprotective role of anti-oxidants in the prevention and treatment of gastric lesions. It is thought that substances with antioxidant properties may protect against the damaging effects of ethanol on the gastric mucosa [1, 4]. Therefore, antioxidants have been proposed as therapeutic agents and drug coadjuvants to prevent gastric injury by inhibiting ROS-induced lipid peroxidation and by increasing the activities of antioxidant enzymes such as catalase (CAT), glutathione S-transferase (GST), glutathione peroxidase (GPx), superoxide dismutase (SOD), and glutathione reductase (GR).

Herbal medicines are used as a supplemental therapy for many kinds of diseases by enhancing the immune responses. The traditional oriental herbal prescription Sipjeondaebo-tang (Shi-Quan-Da-Bu-Tang in Chinese, Juzen-taiho-to in Japanese) comprises 10 different crude components obtained from natural herbs. It is commonly prescribed for the treatment of a depressed or weakened state including fatigue, anemia and anorexia associated with various diseases [5, 6]. Previous experimental reports have shown that Sipjeondaebo-tang exerts various biological activities such as the enhancement of phagocytosis [6], anti-tumor [7], anti-inflammatory [8], and immunomodulatory [9] properties. However, its antioxidant effect has not been studied sufficiently, and there are no reports about whether Sipjeondaebo-tang water extract (SDTW) can prevent ethanol-induced gastric injury. The present study was done to evaluate the antioxidant activities of SDTW against ethanol-induced gastric injury in rats.

Assessing acute toxicity is often the basic step in the study of the safety of a substance and involves rapid methods to measure the concentration that affects the test organisms in a harmful way [10]. Therefore, We performed an acute toxicity study to assess the safety of SDTW.

## Methods

### Preparation of Sipjeondaebo-tang

A voucher specimen of Sipjeondaebo-tang (2008-KE03-1–KE03-5) is available at the Basic Herbal Medicine Research group, Korea Institute of Oriental Medicine. Sipjeondaebo-tang was prepared in our laboratory from a mixture of chopped crude herbs purchased from Omniherb (Korea or China) and HMAX (Vietnam or China). Professor Je-Hyun Lee of Dongguk University, Gyeongju, Republic of Korea, confirmed the identity of each crude herb. Sipjeondaebo-tang was prepared as described in Table 1, and its extract was obtained by boiling the herbs in distilled water at 100°C for 2 h. The solution was evaporated and freeze-dried (yield, 22.9%). A previous study using HPLC analysis identified 10 compounds in Sipjeondaebo-tang: 5-hydroxymethyl-2-furaldehyde, albiflorin, paeoniflorin, liquiritin, ferulic acid, nodakenin, coumarin, cinnamic acid, cinnamaldehyde and glycyrrhizin [11].

**Table 1:** **Crude components of Sipjeondaebo-tang**

Scientific name	Amount (g)	Company of purchase	Source
*Panax ginseng*	3.75 (83%)	Omniherb	Geumsan, Korea
*Cinnamomum cassia*	3.75 (83%)	HMAX	Vietnam
*Cnidium officinale*	3.75 (83%)	Omniherb	Yeongcheon, Korea
*Rehmannia glutinosa*	3.75 (83%)	Omniherb	Pyeongchang, Korea
*Poria cocos*	3.75 (83%)	HMAX	China
*Glycyrrhiza uralensis*	3.75 (83%)	HMAX	China
*Astragalus membranaceus*	3.75 (83%)	Omniherb	Jeongseon, Korea
*Angelica gigas*	3.75 (83%)	Omniherb	Yeongcheon, Korea
*Paeonia lactiflora*	3.75 (83%)	Omniherb	Hwasun, Korea
*Atractylodes japonica*	3.75 (83%)	Omniherb	China
*Zingiber officinale*	3.75 (83%)	Omniherb	Yeongcheon, Korea
*Zizyphus jujuba*	3.75 (83%)	Omniherb	Yeongcheon, Korea
Total amount	45.00 (100%)		

### Ethanol-induced gastric injury

Specific-pathogen-free (SPF) male Sprague Dawley rats weighing 200–250 g (aged 6 weeks) were purchased from Orient Co. (Seoul, Korea) and used after 1 week of quarantine and acclimatization. The animals were kept in a room at 23 ± 3°C with a relative humidity of 50% under a controlled 12 h/12 h light/dark cycle. The rats were given a standard rodent chow and sterilized tap water *ad libitum*. All experimental procedures were performed in accordance with the NIH Guidelines for the Care and Use of Laboratory Animals and were approved by the Korea Institute of Oriental Medicine Institutional Animal Care and Use Committee. The animals were cared for in accordance with the dictates of the National Animal Welfare Law of Korea.

Acute gastric lesions were induced via intragastric administration of absolute ethanol according to a method described previously [12]. The animals were divided into four groups (seven animals in each group): normal control (NC), ethanol (EtOH), omeprazole (Ome), and SDTW (SDTW at 200 mg/kg of body weight) groups. The animals were fasted for 18 h before the experiment. The NC group was given phosphate buffered saline (PBS) by oral gavage (5 mL/kg of body weight) as the vehicle, and the EtOH group was given absolute ethanol (5 mL/kg of body weight) via oral gavage. The Ome group served as a positive control group and was given oral omeprazole (50 mg/kg of body weight) 2 h before the administration of absolute ethanol. Omeprazole is used widely for the treatment of gastritis because of its anti-inflammatory and antioxidant activities [13]. Therefore, it was used as the positive-control drug in the present study. The SDTW group received SDTW (200 mg/kg of body weight) by oral gavage 2 h before administration of absolute ethanol.

The animals were sacrificed via cervical dislocation 1 h after receiving absolute ethanol. The stomach was removed from each animal and opened along its greater curvature. The tissue was rinsed gently in PBS. The stomach was stretched on a piece of cork with the mucosal surface facing upward and was then examined in the standard position for gross examination of gastric mucosal lesions. Photographs of hemorrhagic erosions in the stomach were acquired with a Photometric Quantix digital camera. After the gastric lesions were photographed, the stomach tissue was cut in half and stored at −70°C for biochemical analysis.

### Biochemical analysis

The stomach tissues were cut into small pieces and homogenized (1/10 w/v) with tissue lysis/extraction reagent containing a protease inhibitor (Sigma-Aldrich). The homogenates were centrifuged at 12,000 rpm for 10 min at 4°C to remove any cell debris, and the supernatant was used to measure the concentration of malondialdehyde (MDA), content of reduced glutathione (GSH), and activities of CAT, GST, GPx, SOD and GR. The concentration of total protein was measured using a protein assay reagent (Bio-Rad Laboratories, Inc.).

Lipid peroxidation was estimated by measuring the concentration of MDA using a thiobarbituric acid-reactive substance assay kit (BioAssay Systems, CA, USA). In brief, 100 μL of homogenate was mixed with 100 μL of 10% trichloroacetic acid, and the mixture was incubated for 15 min on ice and then centrifuged at 12,000 rpm for 5 min at 4°C. Two hundred microliters of supernatant was mixed with 200 μL of thiobarbituric acid, and the mixture was incubated at 100°C for 60 min. The absorbance at 535 nm was measured after the mixture was cooled. The results are expressed as μmol/mg protein.

The GSH content was measured using a GSH assay kit (Cayman, MI, USA), and the results are expressed as μmol/mg protein. The activities of the antioxidant enzymes as CAT, GST, GPx, SOD, and GR, were quantified using commercial kits (Cayman) according to the manufacturer’s protocols. The results are expressed as U/mg protein.

### Acute toxicity study

Male and female 5-week-old SD rats were purchased from an SPF facility at the Orient Bio Co. (Seoul, Korea) and used after 1 week of quarantine and acclimatization. All animals were housed in a room maintained at 23 ± 3°C with relative humidity of 50%, artificial lighting from 08:00 to 20:00, and 10–20 air changes/h. The animals were fed a commercial pellet diet (PMI Nutrition International, Richmond, IN, USA) and sterilized tap water *ad libitum* (after UV irradiation and filtration). The acute toxicity study was performed in compliance with the test guidelines of the Korea Food and Drug Administration under the Good Laboratory Practice Regulations for Nonclinical Laboratory Studies [14]. The study protocol was approved by the Institutional Animal Care and Use Committee of the Korea Institute of Toxicology (earned by AALAC International, 1998).

In the preliminary study, a single oral administration of SDTW did not induce any toxicity at a dose up to 5,000 mg/kg. Based on these results, a dose of 5,000 mg/kg was selected as the over-limit dose, than recommended dose by the Organisation for Economic Co-operation and Development test guidelines [15]. Ten rats of each sex were randomly assigned to two groups, with five rats of either sex in each group. The SDTW-administered rats received a single dose of 5,000 mg/kg via oral gavage on day 1 and the vehicle-administered control rats received an equivalent volume of distilled water. Subsequently, all abnormal clinical signs were recorded at least twice a day. Body weight was measured immediately before treatment on the day of dosing (day 1) and on days 2, 4, 8, and 15. At the scheduled termination (day 15), all surviving animals were anesthetized by carbon dioxide and sacrificed by exsanguination from the aorta. A complete gross postmortem examination was performed on all animals.

### Statistical analyses

The Data are expressed as the mean ± standard deviation. Significance was determined using analysis of variance (ANOVA). If the ANOVA showed a significant difference between groups, the data were analyzed further with a multiple-comparison procedure using Dunnett’s test [16]. Statistical analyses were performed using Path/Tox System (Ver. 4.2.2). The level of significance was *p* < 0.05 or 0.01.

## Results

### Effect of SDTW on gastric injury in rats

**Figure 1:**
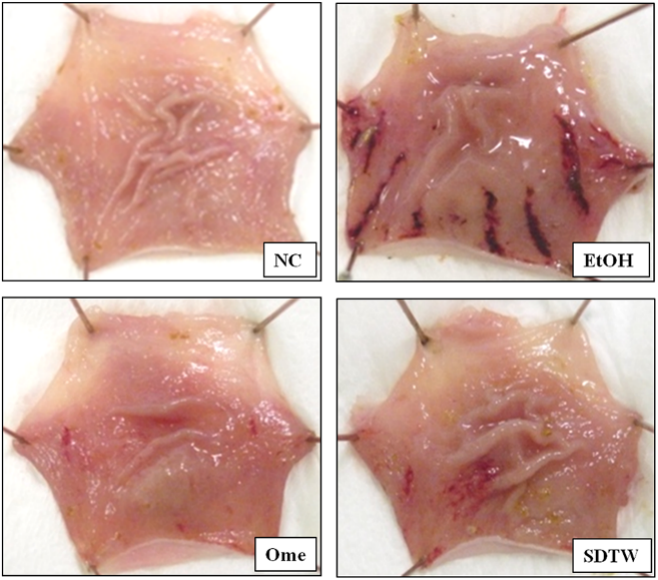
**Gross anatomy of ethanol-induced gastric mucosa injury in rats.** NC: gastric mucosa in the normal control group, EtOH: gastric mucosa in the ethanol-treated group, Ome: gastric mucosa in the group treated with EtOH + omeprazole (50 mg/kg), SDTW: gastric mucosa in the group treated with EtOH + SDTW (200 mg/kg).

Gastric mucosal injury was induced by oral administration of absolute ethanol. Although the EtOH group showed gastric damage, including hemorrhage and hyperemia, the Ome group (positive control) markedly reduced the gastric mucosa injury compared with the EtOH group. SDTW treatment markedly decrease the gastric mucosa injury, and is a similar to Ome groups (Figure 1).

### Effect of SDTW on lipid peroxidation and GSH content

**Figure 2:**
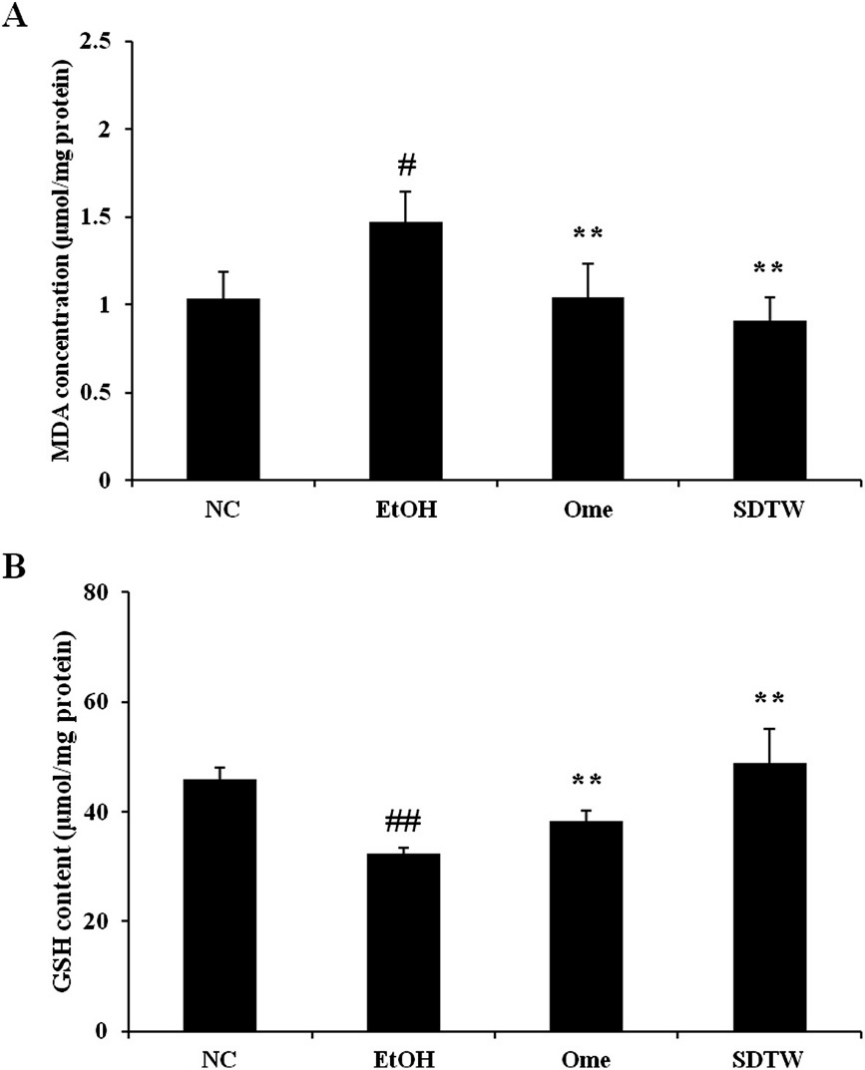
**Effect of SDTW on the malondialdehyde concentration (A) and glutathione content (B) in gastric mucosa.** NC: gastric mucosa in the normal control group, EtOH: gastric mucosa in the ethanol-treated group, Ome: gastric mucosa in the group treated with EtOH + omeprazole (50 mg/kg), SDTW: gastric mucosa in the group treated with EtOH + SDTW (200 mg/kg). The results are expressed as mean ± SD for seven rats. ^##^Significant difference at *p* < 0.01 and ^#^at *p* < 0.05 compared with the NC group. ^**^Significant difference at *p* < 0.01 compared with the EtOH group.

MDA concentration was significantly higher in the EtOH group (1.47 ± 0.17 μmol/mg protein) compared with the NC group (1.04 ± 0.15 μmol/mg protein). By contrast, MDA concentration was significantly lower in the Ome group (1.04 ± 0.19 μmol/mg protein) and the SDTW group (0.91 ± 0.13 μmol/mg protein) compared with the EtOH group (Figure 2A).

GSH content was significant lower (32.30 ± 1.09 μmol/mg protein) in the EtOH group compared with the NC group (45.99 ± 2.05 μmol/mg protein). GSH content was significantly higer in the Ome group (38.30 ± 1.84 μmol/mg protein) and the SDTW group (48.97 ± 6.02 μmol/mg protein) compared with the EtOH group (Figure 2B). Significance was determined using ANOVA. If the ANOVA showed a significant difference between groups, the data were analyzed further with a multiple-comparison procedure using Dunnett’s test. The level of significance was *p* < 0.05 or 0.01.

### Effects of SDTW on antioxidant enzymes activities

The activities of the antioxidant enzymes CAT, GPx, GST, GR and SOD were significantly lower in the EtOH group (238.13 ± 28.07, 55.12 ± 4.83, 8.63 ± 1.34, 57.34 ± 6.06, and 2.39 ± 0.35 U/mg protein, respectively) compared with the NC group (326.23 ± 29.08, 78.54 ± 6.30, 11.19 ± 0.78, 87.84 ± 13.21, and 3.51 ± 0.55 U/mg protein, respectively). CAT and GPx activities were significantly higher in the Ome group (283.55 ± 20.47 and 67.63 ± 7.74 U/mg protein, respectively) compared with the EtOH group. CAT, GPx, GST, and GR activities were significantly higher in the SDTW group (303.38 ± 63.72, 87.20 ± 10.10, 11.54 ± 1.56 and 79.34 ± 10.10 U/mg protein, respectively) compared with the EtOH group. SOD activity was nonsignificantly higher in the SDTW group (2.60 ± 0.49 U/mg protein) compared with the EtOH group (Table 2).

**Table 2:** **Effect of SDTW on the activity of antioxidant enzymes in gastric mucosa**

Groups	Gastric mucosa				
	Catalase activity	GPx activity	GST activity	GR activity	SOD activity
	(U/mg protein)	(U/mg protein)	(U/mg protein)	(U/mg protein)	(U/mg protein)
NC	326.23 ± 29.08	78.54 ± 6.30	11.19 ± 0.78	87.84 ± 13.21	3.51 ± 0.55
EtOH	238.13 ± 28.07^##^	55.12 ± 4.83^##^	8.63 ± 1.34^#^	57.34 ± 6.06^##^	2.39 ± 0.35^##^
Ome	283.55 ± 20.47^*^	67.63 ± 7.74^*^	9.64 ± 0.91	68.16 ± 16.42	2.23 ± 0.61
SDTW	303.38 ± 63.72^*^	87.20 ± 10.10^**^	11.54 ± 1.56^*^	79.34 ± 10.10^*^	2.60 ± 0.49

NC: gastric mucosa in the normal control group, EtOH: gastric mucosa in the ethanol-treated group, Ome: gastric mucosa in the group treated with EtOH + omeprazole (50 mg/kg), SDTW: gastric mucosa in the group treated with EtOH + SDTW (200 mg/kg). The results are expressed as mean ± SD for seven rats. ^##^Significant difference at *p* < 0.01 and at ^#^ *p* < 0.05 compared with the NC group. ^**^Significant difference at *p* < 0.01 and ^*^at *p* < 0.05 compared with the EtOH group.

### Acute toxicity of SDTW

**Figure 3:**
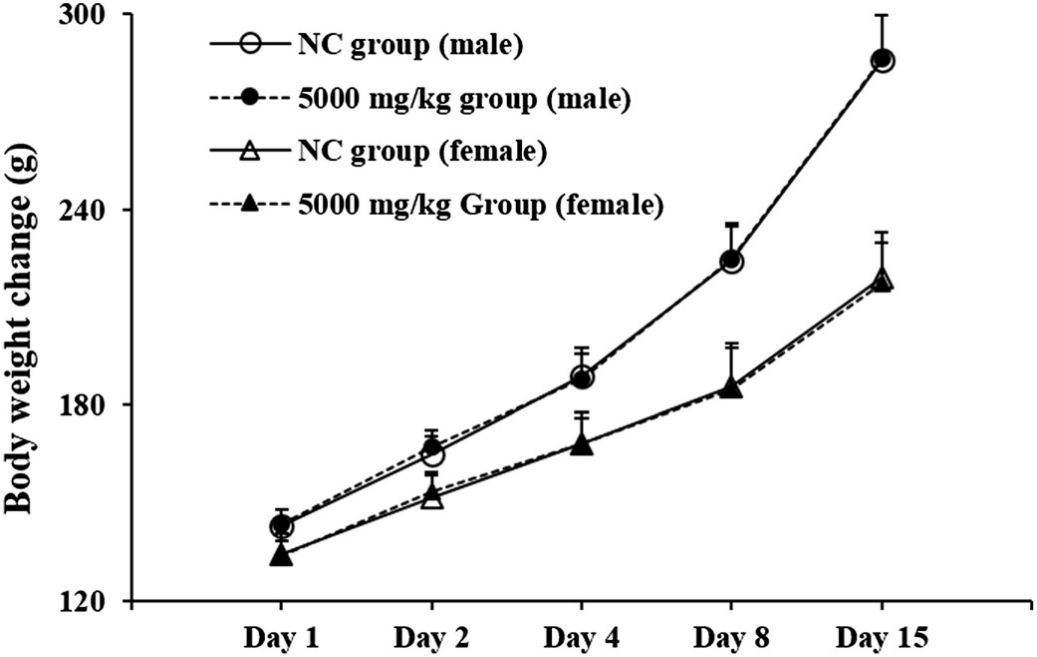
**Body weight changes in acute toxicity.** NC group: normal control group, SDTW-treated group at dose levels of 0 mg/kg (○) and 5,000 mg/kg group: SDTW-treated group at dose levels of 5,000 mg/kg (●) in males and 0 mg/kg (△) and 5,000 mg/kg (▲) in females. There were no significant differences in body weight between the SDTW-treated and NC groups.

We evaluated the acute toxicity of SDTW, to investigate the safety of its oral administration. As shown in Figure 3, there were no significant differences in body weight changes between the SDTW-treated and NC groups for male and female rats. In addition, there were no observed clinical signs or gross findings in the SDTW-treated groups and NC groups (data not shown).

## Discussion

The present study investigated whether SDTW has protective effects against absolute ethanol-induced acute gastric mucosal injury in rats. We found that oral administration of SDTW effectively protected against the acute gastric mucosal injury caused by absolute ethanol. SDTW treatment markedly reduced the gastric damage such as hemorrhage, hyperemia. Pretreatment with SDTW before ethanol administration significantly decreased the MDA concentration and significantly increased GSH content and the activities of antioxidant enzymes compared with treatment with ethanol alone. The acute toxicity study showed that SDTW was safe at a dose of up to 5000 mg/kg.

Traditional herbal medicines have been used in primary health care and to maintain health for thousands of years in some Asian countries. Traditional herbal medicines are a mixture of various herbs, and the effects are improved by interactions between these components [17]. Among the traditional herbal medicines, Sipjeondaebo-tang composed of 10 different herbs and its herbs exhibited various biological effects [18, 19]. However, study on protective effect of Sipjeondaebo-tang on the gastric mucosal injury is rare. Hence, we aimed to determine whether SDTW has antioxidative and anti-inflammatory effects on ethanol-induced gastric mucosal injury.

Absolute ethanol caused linear hemorrhagic lesions and extensive submucosal edema in the stomach, which are typical characteristics of alcoholic injuries [20]. In our study, the ethanol-induced gastric lesions included severe gastric injuries such as hemorrhage and hyperemia, as reported by another study [21]. SDTW treatment markedly attenuated the gastric injury such as hemorrhage and hyperemia. These results suggest that SDTW has a protective action by reducing the gastric mucosal injury exposed to ethanol.

The intake of absolute ethanol is noxious to the stomach. Ethanol causes topical disruption of the gastric mucosal barrier and provokes microvascular changes within a few minutes after its application [22]. Gastric lesions caused by ethanol may be associated with ROS generation; these lesions are caused by an imbalance between oxidant and antioxidant cellular processes [23]. Currently, there is consensus that the harmful effects of ethanol on the gastric mucosa are a consequence of increased lipid peroxidation and a decreased glutathione level [24]. Lipid peroxidation is a major outcome of free-radical-mediated injury, which causes immediate damage to the cell membrane and is related to DNA damage [25]. MDA is a final product of lipid peroxidation and its level is measured as an indication of lipid peroxidation levels in tissues [26]. GSH, the most abundant antioxidant in cells, plays a major role in the defense against oxidative stress-induced cellular injury and is essential for the maintenance of intracellular redox balance [27]. In our study, SDTW treatment significantly decreased MDA concentration and increased GSH content in gastric tissue exposed to ethanol. These results indicate that SDTW has gastroprotective effects against ethanol-induced gastric damage by reducing MDA concentration and increasing GSH content.

A Recent study has shown that ROS are among most the important factors in the pathogenesis of ethanol-induced mucosal injury mediated by oxidative stress [28]. Therefore, gastric injury might be prevented by antioxidants, which may help protect cells from damage caused by oxidative stress and enhance the body’s defense against degenerative diseases. The antioxidant activity may be mediated by inhibition of the formation of radicals or by the scavenging of the formed radicals [29]. The antioxidant enzymes CAT and SOD are believed to play key roles in the enzymatic defense of cells against oxidative stress injury. CAT is a classical oxidative biomarker that exists mainly in peroxisomes of all aerobic cells and serves to protect the cells against damage from hydrogen peroxide [30]. SOD is a metalloenzyme that can convert O_2_ produced during oxidative stress to hydrogen peroxide [31]. GPx, GST, and GR are all glutathione-related enzymes. GPx is an enzyme that plays a fundamental role in the elimination of hydrogen peroxide and lipid hydroperoxides in the gastric mucosa cells [24]. GST can catalyze the conjugation of electrophilic compounds produced during oxidative stress with glutathione [32]. GR is a glutathione-regenerating enzyme that permits the conversion of oxidized glutathione to the reduced form (GSH) [33]. A previous study has demonstrated that antioxidants promote gastroprotection by increasing the antioxidant enzymes activities [34]. We found that the SDTW increased the activities of antioxidant enzymes and reduced ethanol-induced gastric injury. Our results suggest that SDTW can act as an antioxidant as shown by increased the activities of antioxidant enzymes.

Our acute toxicity study showed that SDTW was safe substance when given as a single dose by oral gavage to rats at a dose of 5,000 mg/kg. No clinical signs or gross findings of treatment-related adverse effects were observed in any of the SDTW-treated rats (data not shown).

## Conclusion

In conclusion, the results of this study revealed a gastroprotective role of SDTW against gastric mucosal injury induced by ethanol. The acute toxicity study showed that SDTW (up to 5000 mg/kg) caused no adverse side effects. In addition, SDTW reduced the gastric injuries, increased MDA concentration induced by ethanol, and increased GSH content and activities of the antioxidant enzymes CAT, GPx, GST, GR, and SOD compared with treatment with ethanol alone. The antioxidant activity of SDTW appeared to be mediated through changes in GSH content and the activities of antioxidant enzymes, which may have reduced lipid peroxidation, thereby promoting gastroprotection.
